# Cardiovascular Disease and COVID-19: Insight From Cases With Heart Failure

**DOI:** 10.3389/fcvm.2021.629958

**Published:** 2021-03-15

**Authors:** Yang Yi, Yanan Xu, Haibing Jiang, Jun Wang

**Affiliations:** ^1^Department of Cardiology Fourth Ward, Xinjiang Medical University Affiliated Hospital of Traditional Chinese Medicine, Urumqi, China; ^2^Department of Respiratory Medicine, The People's Hospital of Xuancheng City, Xuancheng, China; ^3^Department of Cardiology, The People's Hospital of Xuancheng City, Anhui, China

**Keywords:** cardiac failure, COVID-19, cardiovascular disease, clinical outcomes, therapeutic strategies, Angiotensin (Ang)-converting enzyme 2

## Abstract

Recent evidence indicates that a large proportion of deaths from coronavirus disease 2019 (COVID-19) can be attributed to cardiovascular disease, including acute myocardial infarction, arrhythmias and heart failure. Indeed, severe infection increases the risk of heart failure among patients with COVID-19. In most patients, heart failure arises from complex interactions between pre-existing conditions, cardiac injury, renin-angiotensin system activation, and the effects of systemic inflammation on the cardiovascular system. In this review, we summarize current knowledge regarding pathogen-driven heart failure occurring during treatment for COVID-19, the potential effects of commonly used cardiovascular and anti-infective drugs in these patients, and possible directions for establishing a theoretical basis for clinical treatment.

## Introduction

In response to the outbreak of COVID-19 in January 2020, China promptly established a national framework for a comprehensive, inter-agency response, which appears to have slowed the spread of the disease and limited the size of the COVID-19 epidemic in China (1). Outside of China, however, the virus has spread widely with the pandemic now reaching almost every country, territory, and region in the world and with high efficiency. For several decades, investigators have noted increases in the short- and long-term risks of major adverse cardiovascular events and mortality due to vascular events following acute respiratory infections, including pneumonia, and a causal relationship has been proposed (2). In the context of COVID-19, increased levels of myocardial cell damage markers such as troponin predict a worse prognosis and appear usually in the middle or later stages of the disease, with some patients displaying extreme elevations in natriuretic peptides and their death being attributed to heart failure (3). From these observations, one hypothesis is that heart failure can be considered a comorbidity or a complication, and in either case, cardiac function has a significant impact on the treatment and prognosis of COVID-19 patients. Observed cardiac complications have ranged from classic heart failure with preserved ejection fraction during the illness's earliest stages to progressive and potentially fatal systolic heart failure occurring later as a response to the cytokine phase of COVID-19. The exact mechanism underlying heart failure in COVID-19 patients remains a matter of discussion, and the most effective approaches for treating cases of COVID-19 with underlying cardiovascular disease remain unclear. Therefore, it is important to obtain a better understanding of how the cardiovascular system is affected by COVID-19, particularly in those with cardiovascular disease, in order to develop strategies for the early detection of cardiac injury and the effective protection of cardiomyocytes in mild and severe cases of COVID-19 in order to prevent or reverse the progression of heart failure in these patients.

## Cardiovascular Disease Among COVID-19 Patients

As the number of COVID-19 cases continues to increase, the number of COVID-19 patients who also have cardiovascular illnesses, such hypertension, coronary artery disease, and heart failure also increases (4, 5). Shi et al. studied 416 hospitalized COVID-19 patients and found that 19.7% of patients had cardiac injury, suggesting an correlation between COVID-19 and cardiac injury (5). Another retrospective cohort study reported that heart failure occurred in 23.0% of 191 patients admitted for COVID-19 (3). It is well-recognized that acute pneumonia can potentially destabilize cardiac function, even in patients without a history of cardiovascular disease. Indeed, studies have provided evidence that severe acute respiratory syndrome (SARS) may also cause acute cardiac injury and chronic damage to cardiac function (6, 7). Because 2019 severe acute respiratory syndrome coronavirus 2 (SARS-CoV-2) and severe acute respiratory syndrome coronavirus (SARS-CoV) have similar pathogenicity, it is also reasonable to hypothesize that they similarly lead to heart failure. Myocardial injury induced by infection with COVID-19 undoubtedly enhances the dilemma and complexity of patient treatment (5). For this reason, it is important clinically to dynamically monitor abnormal laboratory characteristics and echocardiography findings, such as levels of serum troponin and brain natriuretic peptide as well as the left ventricular ejection fraction, in COVID-19 patients, both to detect potential cardiac complications and also to gain an understanding of prevalent abnormalities in COVID-19. In addition, COVID-19 patients may be prone to acute kidney injury after illness onset (3). This observation may support the hypothesis that if renal failure ensues, the ability to process salt and water causes an extra load on the heart and results in a more perilous course with deterioration of cardiac function in heart failure. What is somewhat unique from insights into COVID-19 is that the high frequency of pulmonary complications, noted as bilateral infiltrates on computed scanning, might simply depend on inflammation of viral origin, which leads to exudation, rupture of capillaries and intraparenchymal pulmonary hemorrhagic extravasations, with a high percentage of patients transitioning to hypoxic respiratory failure (3). This prompts the question of whether there is a cardiac contribution to these lung findings and whether an excess volume of congestion results in elevated pressures on the heart and pulmonary arteries that are also in play and being ignored. Further work is required to better understand the mechanisms underlying catheterization with measurement of pulmonary wedge pressure at the beginning of the syndrome and then repeated in the acme phase of clinical expression. To the best of our knowledge, no research has yet examined the occurrence of hypoxia-ischemia and hemodynamics in the context of heart failure in COVID-19 patients.

Importantly, Shi et al. also reported that 40% of confirmed COVID-19 patients that required hospitalization had a history of cardiovascular or cerebrovascular disease, and such underlying disease was closely linked to a higher risk for in-hospital mortality (5). These findings indicate that individuals with underlying cardiovascular disease are both more susceptible to COVID-19 and more likely to experience progression to a critical condition and even death. In reality, several intertwined pathophysiological axes should be discussed, such as increased viscosity during febrile illnesses, heightened coagulation function, proinflammatory effects, and endothelial cell dysfunction, which can all contribute to the predilection for a more perilous COVID-19 infection in those with underlying cardiovascular disease, especially heart failure.

A meta-analysis of eligible studies that included a total of 1,527 COVID-19 patients found that potential complications are likely to increase the risk of death (4). Patients with hypertension and diabetes mellitus were more likely to experience serious disease, with the prevalence rates of these comorbidities being twice as high among serious cases than among non-serious cases, suggesting that hypertension and diabetes mellitus may be risk factors for progression to heart failure among COVID-19 patients (4). Additionally, the prevalence rates of more systemic symptoms and severe pneumonia were higher in patients aged >60 years than in those aged ≤ 60 years, and aging-related immunologic quiescence and proinflammatory effects are more conducive to the early establishment of infection and its negative outcomes (8).

Patients hospitalized for COVID-19 diseases have commonly exhibited non-specific heart palpitations and cardiac arrhythmias (9, 10). Cardiac arrhythmias can trigger new onset heart failure or contribute to worsening heart failure by disrupting synchronization of the cardiac cycle and the coordinated contraction of cardiomyocytes. The high morbidity associated with arrhythmias might be, in part, related to complex interactions between metabolic disarray, hypoxia, and neurohormonal or inflammatory stress in the setting of COVID-19 cases with or without prior cardiovascular disease. Unfortunately, data regarding the types of arrhythmias that occur in COVID-19 patients have yet to be published or presented. Therefore, clinicians should specifically investigate pre-existing cardiovascular disease and symptoms or signs of decompensated heart failure. As the number of new cases continues to increase and more international data become available, analyses of multinational cohorts can contribute to risk stratification for severe disease, particularly for patients with underlying structural heart disease or cardiac insufficiency. Experts should engage in updating the definition and management of advanced heart failure in the setting of COVID-19 at all phases of the illness.

## Heart Failure and Poor Clinical Outcomes in COVID-19 Patients

The Chinese Center for Disease Control and Prevention recently reported that among 44,672 confirmed COVID-19 cases in mainland China, the overall case fatality rate was 2.3%. However, the mortality in patients with cardiovascular disease was much higher at 10.5% (11). Furthermore, according to the largest case series of COVID-19 patients published to date, which had not only a large sample size but also extensive coverage across the geographic regions of China, circulatory diseases remained the most common type of comorbidity and COVID-19 patients with cardiovascular disease accounted for nearly one-third of severe cases and were more likely to experience adverse clinical outcomes (12). These findings were consistent with the strong correlation reported between myocardial injury and death in COVID-19 patients (5). Thus, heart failure in COVID-19 contributes to a poor prognosis. SARS-CoV-2 infection appears to aggravate cardiac dysfunction or induce acute cardiac events in patients with a history of heart disease. Then deterioration of cardiac function complicates the management of COVID-19 in turn. Prior research indicated that SARS-CoV infections led to immune dysregulation and prolonged inflammation that could explain the escalated risk of cardiac diseases in SARS patients (7). Given that SARS-CoV-2 shares a highly similar structure with SARS-CoV, immune dysregulation and prolonged inflammation might be the main causes of poor clinical outcomes in COVID-19 patients. Further longitudinal follow-up data are needed to assess the clinical impact of COVID-19 on future events though. Additionally, studies have indicated that severe reactivation of the Middle East respiratory syndrome-related coronavirus (MERS-CoV) can manifest as potentially fatal acute myocarditis or heart failure, and is associated with a poor prognosis (7, 13). A comparable situation may also occur in COVID-19 patients, considering the similarities in the pathogenicity and myocardial damage induced by SARS-CoV-2 and MERS-CoV. Severe COVID-19 myocarditis could contribute to ejection fraction preserved heart failure (HFpEF), and the typical clinical manifestations in the COVID-19 era may be HFpEF linked primarily to the unmasking of subclinical HFpEF and secondarily to the development of new HFpEF following infection with SARS-CoV-2 (14). The evidence from complete echocardiographic evaluation can be clearly seen in the case of COVID-19 patients, indicating that acute right ventricle dysfunction, possibly related to pulmonary parenchymal or vascular disease, is common, and acute left ventricle systolic dysfunction is not a rare event (15). Therefore, an integrated understanding of the interplay between COVID-19 and cardiac function is needed to support efforts to reduce the risk of heart failure and other cardiovascular events in COVID-19 patients. Follow-up with echocardiography and measurement of myocardial markers and brain natriuretic peptide are recommended after recovery from COVID-19 for patients with heart insufficiency.

## Mechanisms of Heart Failure in COVID-19 Patients

The exact mechanisms underlying heart failure in patients with COVID-19 are not completely understood. Previous studies indicated that SARS-CoV infection of dendritic cells induces low-level expression of antiviral cytokines interferon (IFN)-α and -β, moderate up-regulation of pro-inflammatory cytokines tumor necrosis factor (TNF) and interleukin (IL)-6, and significant up-regulation of the inflammatory chemokines chemokine ligand (CCL)3, CCL5, CCL2, and CXCL10 (16, 17). By comparison, SARS-CoV–infected macrophages also show elevated levels of IFN and other pro-inflammatory cytokines, but these increases are delayed compared with those in dendritic cells (16, 18). Complete genome sequencing of 2019 SARS-CoV-2 established the virus as a novel coronavirus with respect to the sarbecovirus subgenus of the Coronavirus family, the same subgenus as SARS-CoV (17). Additional research indicates that SARS-CoV-2 infection may induce increases in pro-inflammatory factors such as IL-1β, INF-γ, and INF-inducible protein-10 (IP-10) to greater than normal levels during COVID-19 (19). Moreover, patients requiring treatment in the intensive care unit had higher concentrations of IL-2, IL-7, IL-10, granulocyte colony-stimulating factor (GCSF), IP-10, monocyte chemoattractant protein 1 (MCP1), macrophage inflammatory protein 1α (MIP1α), and TNF-α than did those not requiring intensive care unit admission, suggesting that the cytokine storm was associated with disease severity (19). The lungs of COVID-19 patients develop significant pathological lesions, including alveolar exudative inflammation, interstitial inflammation, alveolar epithelium proliferation, and hyaline membrane formation (20). Therefore, abnormal activation of multiple inflammatory pathways may contribute to heart failure triggered by COVID-19, and the cytokine storm is more likely to occur in severe cases requiring ICU treatment. An indirect mechanism for heart failure in COVID-19 mediated *via* cytokine storm can be reasonably hypothesized. Cytokine storm may play a significant role in the progression of cardiomyopathy, or cytokines may contribute to stress-induced myocardial dysfunction in patients with COVID-19.

Angiotensin (Ang)-converting enzyme 2 (ACE2) is a membrane-bound aminopeptidase that plays pivotal roles in both heart failure and pulmonary failure (21–23). Previous studies demonstrated that SARS-CoV infection in mouse lungs causes ACE2-dependent myocardial infection (22, 23). Recent research found that SARS-CoV-2 has a 10- to 20-fold greater binding affinity to ACE2 compared with SARS-CoV, which may explain how SARS-CoV-2 is so easily spread from person-to-person (20). Accordingly, extra attention should be paid to applying strategies for cardiovascular protection during treatment of COVID-19. Interestingly, the protective role of the ACE2/Ang-(1–9) axis is related to cardiovascular remodeling. Under normal physiological conditions, the activity levels of the positive ACE/Ang II axis and negative ACE2/Ang-(1–7) axis of the renin-Ang system (RAS) are in a dynamic equilibrium, which maintains the normal function of the cardiovascular system, including dilating blood vessels, lowering blood pressure, and inhibiting apoptosis (3). Overexpression of ACE2 enhances plaque stability in a rabbit model of atherosclerosis (24). Furthermore, ACE2 overexpression and Ang (17) significantly improve ventricular remodeling and function in a rat model of myocardial infarction (25, 26). Similarly, clinical studies have demonstrated that plasma Ang-(1–7) levels in patients with acute myocardial infarction are significantly correlated with myocardial survival index, myocardial infarction area and left ventricular ejection fraction after coronary intervention (27). Previous findings support a significant effect of overexpression of ACE2 and plasma Ang-(1–7) for reducing the incidence and severity of abdominal aortic aneurysm major (28). Notably, this pathway is grossly perturbed by SARS-CoV-2 infection (29, 30), which may result in a decline in ACE2 levels and elevation of plasma Ang-(1–7) levels. The excessive inflammation induced by increased pro-inflammatory factor expression levels may result in a cytokine storm, which contributes to myocardium damage (29, 30). However, ACE2 up-regulation and highly regulated tissue injury are found in patients with pre-existing cardiomyopathy and other underlying diseases of myocardial injury (31, 32), which may facilitate the invasion of SARS-CoV-2 into the body. Therefore, patients with cardiomyopathy are more likely to be experience severe COVID-19. *In vitro* experiments and studies in animal models revealed that the mechanism by which SARS-CoV activates the RAS positive axis is *via* downregulation of ACE2 levels, which in turn triggers acute severe lung injury (21, 33). Also noteworthy is that ACE2 is highly expressed in the heart and lungs, but SARS-nCoV-2 mainly affects the alveolar epithelium with only a minimal effect on the heart (34). ACE inhibitor (ACEI) and Ang II receptor antagonist (ARB) can increase the expression of ACE2 or prevent the loss of ACE2, effects that contribute to the mechanisms of ACEI/ARB activity. Accordingly, it could be reasonably hypothesized that ACEIs/ARBs might increase the risk of SARS-CoV-2 infection or induce increased expression of ACE2 in the cardiovascular system and lung tissue, which would aggravate the condition. However, this phenomenon has not been observed clinically (35). One explanation may be that SARS-CoV-2 rarely invades the blood circulation, limiting viremia. Therefore, the virus may not cause myocardial damage directly through the ACE2 pathway. Other explanations may be that the so-called ACE2 proteins of the heart and lungs are slightly different subtypes or that SARS-CoV-2 does not function through ACE2 at all. Importantly, biological experiments have shown that the severe phenotype of mice with a single mutation of Ace2 can be rescued by ACE deficiency resulting from further deletion of the Ace gene17, indicating that the balance of ACE2/ACE levels is the vital target for preventing lung injury and achieving lung protection (23).

## Therapeutic Strategies for Heart Failure in COVID-19 Patients

SARS-CoV-2 invades the human body in the same way as SARS-CoV by binding to Spike protein, which causes down-regulation of tissue ACE2 expression and elevation of AngII expression (20, 36, 37). Given that both the heart and lung are organs with high expression of ACE2 and share a common pathogenic basis, theoretically the heart and lung should be almost similarly affected by SARS-CoV-2 infection (36, 37). In a previous study of consecutive healthy humans treated with ACEIs and controls, the mean level of duodenal ACE2 mRNA expression was increased by 1.9-fold compared with that in non-treated controls, and interesting, no significant differences in ACE2 mRNA expression levels were observed during treatment with ARBs (38). Whether improved ACE2 expression with ACEI/ARB use increases the susceptibility to COVID-19 remains to be determined. Notably, ACE2 expression is a dominant mechanism for negative regulation of RAS conversion of Ang II into the beneficial peptide Ang 1–7, and this significant biochemical and physiological property is being harnessed as a potential therapy for patients with heart failure (39). However, emerging data suggest that ACEIs, such as captopril and lisinopril, do not affect ACE2 activity, and there continues to be no direct evidence of a connection between ACE2 expression and the susceptibility to and severity of SARS-CoV-2 infection (29). A previous meta-analysis also showed that ACEIs/ARBs may have a protective effect in patients with community-acquired pneumonia and may reduce pneumonia-related mortality compared with that observed for the control group (30). Of note, losartan has been shown to ameliorate lung injury caused by SARS-CoV in an experimental study (40, 41). Therefore, losartan may be a possible treatment method for reducing acute cardiopulmonary injury that can be applied in the clinic immediately (40, 41). Unfortunately, the effects of ACEIs/ARBs in the treatment of patients with heart failure during COVID-19 have yet to be established.

Moreover, betablockers, as another major class of drugs used in heart failure treatment, may have beneficial effects in COVID-19 patients through several functions, ranging from reducing adrenergic activation (like their pivotal role in Takotsubo cardiomyopathy), inhibiting NLRP3 inflammasome, and decreasing SARS-CoV-2 entry into the cell by downregulating ACE2 receptors (still only a hypothesis) (42). Spironolactone may also provide protection through concurrent actions in the modulation of ACE2 expression and increasing angiotensin 1–7 levels (43). Moreover, as an anti-androgenic agent, it can decrease viral priming through TMPRSS2 activity (44). Therefore, spironolactone seems to be an ideal drug candidate that can attenuate the harmful effects of the overexpression of angiotensin II-AT-1 axis, and more importantly, is pharmacologically safe in the treatment of COVID-19 patients with heart failure.

Based on the above possible mechanisms of the interrelationship between the adrenergic system, RAAS, and ACE2, novel strategies proposed for the prevention and treatment of heart failure in patients with COVID-19 are of significance. A drug with little effect on the expression and activity of the ACE2/Ang-(1–7) axis may be beneficial. Recently, the efficacy and safety of the sodium–glucose co-transporter 2 (SGLT2) inhibitor dapagliflozin has been demonstrated for a broad spectrum of patients with heart failure and reduced ejection fraction, with only a minimal effect on ACE2 expression (44). The first study to analyze nationwide data of 1,590 COVID-19 cases in China reported that the prevalence rates of specific comorbidities were 16.9% for hypertension (*n* = 269), 8.2% for diabetes (*n* = 130), and 1.3% for chronic kidney diseases (*n* = 21) (11). Given the benefits of SGLT2 inhibitors for renal function, the effects of type 2 diabetes on heart failure, and the prevalence and importance of renal impairment in heart failure with reduced ejection fraction (45, 46), it is possible that treatment with the SGLT2 inhibitor dagliflozin may enhance the effect of drug therapy, reducing complications and minimizing the possible negative effects of drugs in COVID-19 patients with heart failure. Moreover, SGLT2 inhibitors were shown to not only reduce systolic blood pressure but also diastolic blood pressure (47). Interestingly, this antihypertensive effect of SGLT2 inhibitors does not lead to an increase in heart rate compensation, indicating that no compensatory sympathetic activation occurs (48). Animal experiments showed that SGLT2 inhibitors can reduce hyperglycemia-induced leukocytosis and inhibit the inflammatory response and oxidative stress (49). SGLT-2 inhibitors, irrespective of the status of blood glucose control, decrease the levels of pro-inflammatory cytokines (50). To summarize, among the medication options for COVID-19 complicated with heart failure, the inclusion of new medicines such as SGLT2 inhibitors may help to maximize the efficacy of anti-heart failure treatments and minimize the negative impacts of other drugs on heart function. However, any new treatment options for COVID-19 patients with severe disease should be examined in the context of randomized clinical trials. The safety and reliability of SGLT2 inhibitors need to be established in more large-scale clinical studies. Additionally, perhaps the most important finding will guide the field of SGLT2 inhibitors in organ protection beyond viral infection or diabetes (51). Moreover, the most frequent abnormality was right ventricle dilation with or without dysfunction in patients with COVID-19 (15), and SGLT2 plays a role in reducing right ventricle systolic pressure (52). Therefore, we can hypothesize that this drug may play a pivotal role in the prevention of pulmonary edema and reducing pulmonary wedge pressure.

## Conclusions

Several pathogen-driven mechanisms of COVID-19 pneumonia have been proposed and could serve as targets for future therapeutic interventions to reduce the incidence of heart failure and improve outcomes in patients with COVID-19. COVID-19 should be recognized as more than a respiratory disease and more appropriately as a systemic disease. Meticulous attention to potentially fatal complications of COVID-19 and abnormal laboratory characteristics is necessary to enable early diagnosis and appropriate treatment of such complications. Whether treatment with specific medicines or disease-induced down-regulation of ACE2 exacerbates the progression of COVID-19 urgently needs to be determined.

## Data Availability Statement

The original contributions presented in the study are included in the article/supplementary material, further inquiries can be directed to the corresponding author.

## Author Contributions

JW was responsible for the overall content as the guarantor and conceived and designed the review. YY and YX wrote the paper. HJ: revision. The manuscript was approved by all authors.

## Conflict of Interest

The authors declare that the research was conducted in the absence of any commercial or financial relationships that could be construed as a potential conflict of interest.
